# Four Models Including Fish, Seafood, Red Meat and Enriched Foods to Achieve Australian Dietary Recommendations for *n*-3 LCPUFA for All Life-Stages

**DOI:** 10.3390/nu7105413

**Published:** 2015-10-19

**Authors:** Flavia Fayet-Moore, Katrine Baghurst, Barbara J. Meyer

**Affiliations:** 1 Nutrition Research Australia, Level 13/167 Macquarie St, Sydney, NSW 2000, Australia; 2 CSIRO and consultant, Adelaide, SA 5000, Australia; 3 School of Medicine, University of Wollongong, Northfields Ave, Wollongong, NSW 2522, Australia; bmeyer@uow.edu.au

**Keywords:** omega-3 long chain polyunsaturated fatty acids (*n*-3 LCPUFA), recommended intakes, suggested dietary target intakes, omega-3 (*n*-3) enriched foods, dietary modelling

## Abstract

Populations are not meeting recommended intakes of omega-3 long chain polyunsaturated fatty acids (*n*-3 LCPUFA). The aim was (i) to develop a database on *n*-3 LCPUFA enriched products; (ii) to undertake dietary modelling exercise using four dietary approaches to meet the recommendations and (iii) to determine the cost of the models. Six *n*-3 LCPUFA enriched foods were identified. Fish was categorised by *n*-3 LCPUFA content (mg/100 g categories as “excellent” “good” and “moderate”). The four models to meet recommended *n*-3 LCPUFA intakes were (i) fish only; (ii) moderate fish (with red meat and enriched foods); (iii) fish avoiders (red meat and enriched foods only); and (iv) lacto-ovo vegetarian diet (enriched foods only). Diets were modelled using the NUTTAB2010 database and *n*-3 LCPUFA were calculated and compared to the Suggested Dietary Targets (SDT). The cost of meeting these recommendations was calculated per 100 mg *n*-3 LCPUFA. The SDT were achieved for all life-stages with all four models. The weekly food intake in number of serves to meet the *n*-3 LCPUFA SDT for all life-stages for each dietary model were: (i) 2 “excellent” fish; (ii) 1 “excellent” and 1 “good” fish, and depending on life-stage, 3–4 lean red meat, 0–2 eggs and 3–26 enriched foods; (iii) 4 lean red meat, and 20–59 enriched foods; (iv) 37–66 enriched foods. Recommended intakes of *n*-3 LCPUFA were easily met by the consumption of fish, which was the cheapest source of *n*-3 LCPUFA. Other strategies may be required to achieve the recommendations including modifying the current food supply through feeding practices, novel plant sources and more enriched foods.

## 1. Introduction

There is a growing body of evidence worldwide that the consumption of omega-3 long-chain polyunsaturated fatty acids (*n*-3 LCPUFA), namely eicosapentaenoic acid (EPA), docosapentaenoic acid (DPA) and docosahexaenoic acid (DHA), is associated with numerous health outcomes, specifically in cardiovascular disease prevention [1,2]. The National Health and Medical Research Council (NHMRC) has set Nutrient Reference Values (NRV) for *n*-3 LCPUFA [3], which differ by life-stage and gender. The NHMRC Suggested Dietary Targets (SDT) is defined as “A daily average intake from food and beverages for certain nutrients that that may help in prevention of chronic disease”. The SDT apply to adults and adolescents 14 years and over and the SDT for *n*-3 LCPUFA are set at 610 mg/day for men and 430 mg/day for women. The International Society for the Study of Fatty Acids and Lipids (ISSFAL) and the National Heart Foundation of Australia (NHFA) recommends that all Australians consume 500 mg of *n*-3 LCPUFA per day to lower the risk of heart disease [4,5]. Other countries recommend the consumption of two fish meals per week which is equivalent to 500 mg *n*-3 LCPUFA per day [6,7,8,9,10,11].

In recognition of the importance of DHA during pregnancy and lactation, the European Consensus statement [12], the World Health Organization guidelines [13] and a European consensus statement [14] all recommend 200 mg DHA/day for pregnant women, whilst the International Society for the Study of Fatty Acids and Lipids (ISSFAL) recommend 300 mg DHA during pregnancy and lactation [4]. Despite the international recommendation for DHA during pregnancy, many pregnant women lack the understanding and knowledge of the importance of DHA [15] and also do not meet the recommendation of 200 mg DHA per day [16]. A useful pamphlet has been developed specifically designed to increase awareness of DHA for pregnant women and consequently increase *n*-3 LCPUFA intake [17].

A small study in the Illawarra region of New South Wales, Australia showed that the median intake of *n*-6 and *n*-3 PUFA was 9.9 g and 1.2 g per day respectively [18]. Similar results were found from the National Nutrition Survey (NNS, *n* = 13,858) where the intakes of *n*-6 and *n*-3 PUFA were 10.9 and 1.36 g per day respectively [19], showing that Australians consume 8 times more *n*-6 PUFA than *n*-3 PUFA. Furthermore, the linoleic acid intakes were 10.64 g per day and the DHA intakes were only 0.1 g per day [20]. A study by Lassek *et al.* showed that DHA from breast milk was positively associated with cognitive performance, whilst LA from breastmilk was negatively associated with cognitive performance [21], suggesting that increased consumption of *n*-3 PUFA including DHA is warranted. The main dietary source of *n*-3 LCPUFA is fish/seafood (66%), followed by meat/poultry/game (29%) and eggs (5%) [18], with similar results from the NNS, with fish/seafood (71%), meat/poultry/game (20%) and eggs (6%) the major contributors to *n*-3 LCPUFA intakes [19]. Given that meat/poultry/game contributed at least 20% to *n*-3 LCPUFA intakes, this NNS was re-analysed after analytical fatty acid data became available on meat [22]. The re-analysed NNS showed that the previous reports under-estimated the contribution of meat to the *n*-3 LCPUFA intakes, as meat contributed close to 50% of *n*-3 LCPUFA intakes [20,23]. This is not because meat itself is a rich source of *n*-3 LCPUFA but because Australians consume at least 7 times more meat than fish/seafood [18,19,20]. Concurrently, recent reports on consumption show that Australians are not meeting *n*-3 LCPUFA recommendations [19,24,25,26]. Hence, there is a need to explore more practical options of achieving the recommended *n*-3 LCPUFA intake that provide consumers with a range of food-based choices to meet their dietary needs.

Although some groups have suggested the use of *n*-3 LCPUFA enriched foods [4,27,28,29] to meet recommendations, none have specified amounts of *n*-3 LCPUFA-enriched foods and beverages that need to be consumed to meet recommendations and that are commercially available. Food-based guidelines for meeting the recommended target of 500 mg per day of combined docosahexaenoic acid (DHA) and eicosapentaenoic acid (EPA) typically focus on: “*2–3 serves (150 g) of oily fish per week*” [5]. Whilst enriched foods and drinks are mentioned in recommendations, there is little dietary information available for consumers on alternative ways to meet daily or weekly *n*-3 LCPUFA intake. Despite many food products being enriched with *n*-3 LCPUFA in Australia, there is no comprehensive, up-to-date database on these foods.

Therefore, the aim of this study was (i) to develop a database on *n*-3 LCPUFA enriched foods and beverages; (ii) to undertake dietary modelling exercises using four dietary approaches to meet the nationally-set recommended *n*-3 LCPUFA intake for different life-stages; and (iii) to determine the cost of obtaining 100 mg of *n*-3 LCPUFA from the different food sources.

## 2. Experimental Section

### 2.1. Database

A supermarket trawl was undertaken to identify all *n*-3 LCPUFA enriched products available from four supermarkets in the metropolitan areas of Wollongong and Sydney in New South Wales, Australia. All *n*-3 LCPUFA enriched products were identified and the full name of the food, serving size, energy, macronutrient and total polyunsaturated fat, total *n*-6, total *n*-3, alpha-linoleic acid (ALA), EPA, DHA and total *n*-3 LCPUFA were recorded where available. In addition, the *n*-3 source was noted and cost per serve, as well as per 100 g, was calculated. A total of six products were identified and used in the modelling (see Appendix Table A1). These products included *n*-3 LCPUFA enriched bread, eggs, yoghurt, milk, flavoured beverage powder, and almond meal.

### 2.2. Models

Foods were included in the model if they met the Food Standards Australia New Zealand (FSANZ) Food Standards Code for voluntary *n*-3 fatty acid nutrition claims [30]. A claim that a food is a “source” of *n*-3 PUFA must contain no less than 200 g of ALA or 30 mg total EPA and DHA per serve, while a “good source” must contain no less than 60 mg total EPA and DHA per serve. Docosapentaenoic acid (DPA) is not included in the content claim recommendations, even though it contributes to *n*-3 LCPUFA intakes [2,19,23]. Serving sizes were consistent with National Dietary Guidelines for Australians [31] and set at 100 g of cooked fish or meat.

Diets were modelled using the NUTTAB2010 foods database [32]. Three subgroups of fish were developed based on *n*-3 LCPUFA content. Fish was categorized by *n*-3 LCPUFA content as “excellent” (≥1200 mg/100 g), “good” (200–1200 mg/100 g) and “moderate” (<200 mg/100 g) (Table 1).

The average *n*-3 LCPUFA content of all meats were calculated using NUTTAB2010 by averaging *n*-3 LCPUFA content of all cooked lean cuts of meat. Red meat (beef, lamb, veal) and pork, met the FSANZ source claim of at least 30 mg of DHA and EPA per serve (100 g). Pork and chicken were not used in the dietary modelling due to their lower *n*-3 LCPUFA content compared to red meat. The average *n*-3 LCPUFA (DHA, EPA, DPA) for red meat, including beef, lamb and veal, was set at 119 mg/100 g, and a cut with approximately 119 mg/100 g serve was used in the model (e.g., beef mince, lamb shanks, scotch fillet).

**Table 1:** Types of fish and red meat used in the model categorized by content of *n*-3 LCPUFA.

Excellent Source of *n*-3 LCPUFA Fish	Good Source of *n*-3 LCPUFA Fish	Moderate Source of *n*-3 LCPUFA Fish and Red Meat
*≥1200 mg/100 g*	*200–1200 mg/100 g*	*<200 mg/100 g*
Salmon, trout, silver perch, canned salmon (pink or red)	Smoked salmon, bream, anchovy, mullet, tinned tuna, snapper, flathead, calamari/squid, oysters, mussels	Barramundi, whiting, tilapia, prawn, fish fingers, shark (flake), fish cake, fish battered Beef, lamb, veal

Four dietary models were developed and calculated based on weekly intake. Model 1 included fish/seafood only (high fish consumers) equivalent of 2–3 “excellent/moderate” LC *n*-3 fish serves/week. Model 2 included some fish (moderate fish consumers) and equivalent to a maximum of 1 “excellent” LC *n*-3 fish serve/week in addition to lean red meat, eggs and *n*-3 LCPUFA enriched foods. Model 3 did not include fish, but did include lean red meat, eggs and *n*-3 LCPUFA enriched foods (non-fish consumers). Model 4 included only *n*-3 LCPUFA enriched foods (suitable for lacto-ovo vegetarian diets). Within each model, serves of red meat and fish as well as dairy and eggs were modelled based on the National Dietary Guidelines for Australians. Red meat was maximized at 3–4 weekly serves, fish at 2–3 weekly serves, dairy at three serves per day, eggs at six per week and bread at up to six slices or three serves per day.

### 2.3. Recommended Intake

The total *n*-3 LCPUFA in each model was calculated and compared to the NHMRC NRV SDT [3]. The SDT was multiplied by seven to calculate weekly target intakes. The NHMRC have SDT for *n*-3 LCPUFA for 14 year olds and older males at 610 mg/day and for females at 430 mg/day [3]. For the purposes of this modelling exercise, the SDT were adjusted for other age groups based on the age and sex-specific energy intakes for each age group [25]. For example, mean energy intake for 2–3 year old boys was 53% of that of 14–16 year olds [20], and hence the adjusted SDT (aSDT) for *n*-3 LCPUFA for 2–3 year old boys (323 mg/day) was set at 53% of the SDT for 14–16 year olds (610 mg/day). The other age groups were calculated according to their proportion of energy intake. Similar calculations were carried out for the various age groups for girls, using 430 mg per day for the 14–16 year old girls [26].

### 2.4. Cost Analysis

Average costs per pack in Australian dollars, per 100 g and per serve were recorded during the supermarket trawl and obtained online. The price for each popular lean cut of meat was obtained for supermarket-branded meats and averaged for beef, lamb and veal; lean red meat was calculated using the ratio of (beef + veal): lamb, or 4.3:1. Cost per 100 g (cooked weight) of lean red meat was then calculated. For fish and seafood, the average price of fresh, frozen and tinned/processed forms were obtained. The cost of obtaining 100 mg of *n*-3 LCPUFA for each diet modelled was calculated.

*n*-3 LCPUFA values for classification into “excellent”, “good” and “moderate” were taken from NUTTAB 2010 (http://www.foodstandards.gov.au/consumerinformation/nuttab2010/).

## 3. Results

All four dietary models were able to meet the SDT for all life stages (Table 2). In model 1 the recommended SDT were easily met for all life-stages with two serves of “excellent” sources of *n*-3 LCPUFA (Table 1). At three serves of fish per week, SDT were met if one serve was an “excellent” source (Table 1) and the other two serves were “good” sources. Specifically for children, the SDT were met by a combination of “excellent” and “moderate” *n*-3 LCPUFA fish sources. For example, a 2–3 year old child could consume 100 g tinned salmon (an “excellent” source), and 100 g tinned tuna (a “good” source) to meet their weekly SDT.

In model 2 (Table 2), the type of fish was maximised at one serve of “excellent” fish per week and, therefore, a weekly intake of one “moderate” or “good” source of fish, four red meat serves (maximum serves per dietary guidelines) and the inclusion of three to 26 weekly serves of *n*-3 LCPUFA enriched foods (including enriched and non-enriched eggs) was necessary to meet the SDT recommendations. For children, where the SDT is lower than adults (see methods), a combination of “good” and “moderate” sources of fish was sufficient. The SDT for all children could be met with either: two fish serves per week (a “good” and a “moderate” source) and meat and enriched foods; or one serve of fish per week (an “excellent” source) and enriched foods. For 2–3 year old children as an example, 100 g of crumbed fish cake made with salmon (a “good” source), 100 g crumbed fish fingers (a “moderate” source), 300 g of mince, 3 *n*-3 LCPUFA enriched eggs and 7 × 90 g *n*-3 LCPUFA enriched yoghurts would be needed per week to meet their weekly SDT. For adult males using model 2, the SDT was reached with two serves of fish (an “excellent” and a “moderate” source), 400 g of lean beef, six enriched eggs, eight cups of milk and 10 slices of bread per week.

In model 3 (Table 2), where individuals do not consume fish, the maximum recommended four serves of red meat intake was necessary to meet the SDT for all life stages. All egg intake had to be *n*-3 LCPUFA enriched and up to 59 weekly serves, or about 8 serves per day of *n*-3 LCPUFA enriched products per day were also necessary. For a 2–3 year old child, this translates to 400 g mince, 6 *n*-3 LCPUFA enriched eggs and 3 cups of *n*-3 LCPUFA enriched milk a week, and approximately two slices of *n*-3 LCPUFA enriched bread and just over 100 g of *n*-3 LCPUFA enriched yoghurt per day. A sample diet to meet the SDT for adults of all life stages would be 400 g beef, six enriched eggs, two kilograms of yoghurt, 28 slices of bread and 11 cups of milk per week.

**Table 2:** Weekly food intake to meet the Suggested Dietary Target (SDT) *n*-3 LCPUFA intakes for all life-stages (2y+).

Food	Model 1		Model 2		Model 3		Model 4	
	*Fish Only*		*Moderate Fish*		*No fish*		*Vegetarian*	
	Serves	Sample Diet	Serves	Sample Diet	Serves	Sample Diet	Serves	Sample Diet
Fish	2 serves of “excellent” fish	100 g Atlantic salmon 200 g fish cake made with salmon	1 serve of “excellent” fish plus 1 serve of “good” or “moderate” fish	100 g Atlantic salmon 100 g King prawns	Nil	Nil	Nil	l
Lean red meat	Nil	Nil	3–4 serves	400 g beef	4 serves	400 g beef	Nil	Nil
Eggs ^1^	Nil	Nil	0–2 serves	Nil	Nil	Nil	Nil	Nil
*n*-3 LCPUFA enriched foods ^2^	Nil	Nil	3–26 serves	6 eggs 10 slices bread 8 cups milk	20–59 serves (3–8 serves per day)	6 eggs 21 × 90 g tubs of yoghurt 28 slices bread 10.5 cups milk 7 serves beverage powder	37–66 serves (5–9 serves)	6 *n*-3 eggs 21 × 90 g tubs of yoghurt 28 slices bread 2 Friands made with DHA almond meal 16 cups milk 7 serves of beverage powder

Model 1—2–3 serves of fish serves per week only (no red meat or *n*-3 LCPUFA enriched foods); Model 2—Maximum of 1 “excellent” source of *n*-3 LCPUFA fish per week with red meat and *n*-3 LCPUFA enriched foods; Model 3—Red meat and *n*-3 LCPUFA enriched foods only (no fish); Model 4—*n*-3 LCPUFA enriched foods only suitable for lacto-ovo vegetarians. ^1^ Whole non-enriched eggs; ^2^ Includes *n*-3 LCPUFA enriched eggs.

Model 4, the lacto-ovo vegetarian diet (Table 2) excludes two significant sources of *n*-3 LCPUFA, namely fish and red meat (beef/veal/lamb) and therefore the remaining source of *n*-3 LCPUFA in the diet included only *n*-3 LCPUFA enriched foods and eggs, which are maximised at six eggs per week by the National Heart Foundation recommendation. In order to meet the SDT for all life stages, a minimum of 37 and a maximum of 66 *n*-3 LCPUFA enriched food serves need to be consumed in one week. This translates to all milk, bread and egg consumption to be in the *n*-3 LCPUFA enriched form. An adult would need to consume enriched foods in model 3 plus 2 Friands (a type of muffin traditionally made with almond meal and popular in Australia) made with DHA almond meal and 5.5 cups of milk per week to meet their SDT.

**Figure 1:**
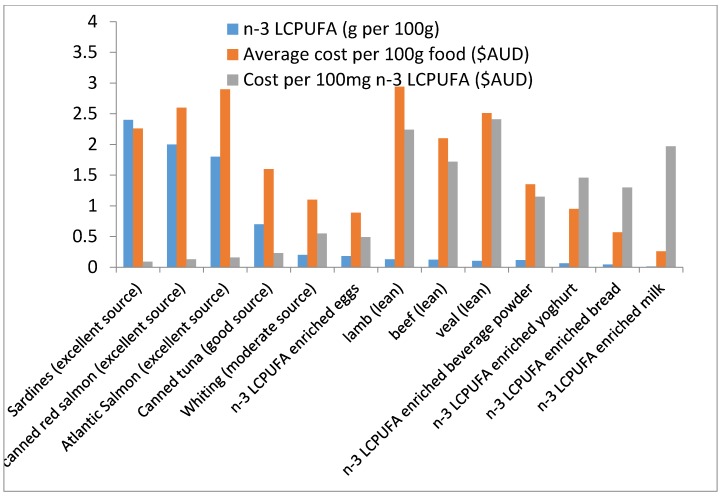
The amount of *n*-3 PUFA in fish and enriched foods, average cost * per 100 g of food and per 100 mg of *n*-3 LCPUFA. (* Cost estimated in December 2012.) Almond meal was excluded from the figure due to very low *n*-3 LCPUFA content.

The amount of *n*-3 LCPUFA (g per 100 g), the average cost per 100 g of food (fish or enriched food) and the cost per 100 mg of *n*-3 LCPUFA is shown in Figure 1. Excellent sources of fish (sardines and salmon) are amongst the most expensive foods but they provide the most *n*-3 LCPUFA and hence are the least expensive when expressed as cost per delivery of 100 mg *n*-3 LCPUFA. Lean meats are comparable in cost to excellent sources of fish but contain far less *n*-3 LCPUFA than fish and therefore are amongst the highest cost per delivery of 100 mg *n*-3 LCPUFA. The *n*-3 enriched foods have very low levels of *n*-3 LCPUFA per 100 g of food and hence the cost per delivery of 100 mg *n*-3 LCPUFA is much higher than fish. Excellent sources of fish provide the greatest amount of *n*-3 LCPUFA and the cost per 100 mg *n*-3 LCPUFA is the least (Figure 1). Fish oil supplements also provide high levels of *n*-3 LCPUFA and the cost per delivery of 100 mg *n*-3 LCPUFA is approximately 0.04 AUD. Unlike supplements that only provide vitamin E, the foods provide other nutrients in addition to *n*-3 LCPUFA: fish provides selenium, iodine, zinc; eggs provide iodine, selenium and biotin; meats provide iron, vitamin B_12_ and zinc; yoghurt and milk provide calcium; and bread provides fibre.

## 4. Discussion

The nutritional intakes of *n*-3 LCPUFA (and DHA) of Australian men is 0.298 g per day (0.117 g per day); of Australian women is 0.195 g per day (0.083 g per day); of Australian elderly people is 0.219 g per day (0.096 g per day) [20] and of pregnant women 0.263 g per day (0.099 g per day) [16]. The SDT for *n*-3 LCPUFA were achieved for all life-stages with all four dietary models. The weekly food intake to meet the *n*-3 LCPUFA SDT for all life-stages for each dietary model was: 2 serves of an “excellent” source of *n*-3 LCPUFA fish (Model 1), 1 serve of an “excellent” and 1 serve of a “good” source of *n*-3 LCPUFA fish, 3–4 serves of lean red meat, 0–2 serves of eggs and 3–26 serves of *n*-3 LCPUFA enriched foods (Model 2), 4 serves of lean red meat, and 20–59 serves of *n*-3 LCPUFA enriched foods (Model 3), 37–66 serves of *n*-3 LCPUFA enriched foods (Model 4).

In the Australian diet, the median *n*-3 LCPUFA intake in adults (*n* = 10,851) was approximately 125 mg/day [22], which is well under the target of 500 mg/day [5]. A more recent Australian National Nutrition Survey conducted in 4487 children aged 2–16 years old showed that the median intakes ranged from 56 mg/day (2–3 years old) to 98 mg/day (14–16 years old) and only 6% of children met the SDT for *n*-3 LCPUFA per day [26]. Therefore, there is a need to increase *n*-3 LCPUFA in the diets of Australian adults and children in order to meet the SDT.

Fish/seafood, meat, eggs, *n*-3 LCPUFA enriched products and supplements are virtually the only sources of *n*-3 LCPUFA in Australia. Despite fish and seafood being the richest source of *n*-3 LCPUFA, they are not widely consumed by adults and children [33]. Based on the 1995 National Nutrition Survey and the 2007 Australian National Children’s Nutrition and Physical Activity, the mean daily fish consumption in Australia was approximately 27 g for adults [33] and 13 g for children, whilst the median fish intake was zero [26]. Only 20% of children consumed fish or seafood and of the children that did consume fish and seafood, these children were originally born in countries where fish/seafood is traditionally eaten, like Japan, Korea and the Seychelles [26]. However the vast majority of Australians consume meat in quantities at least 7 times greater than fish/seafood [18,19,20,26], hence meat has been shown to be a major contributor of *n*-3 LCPUFA intake (47%) to the Australian diet [19,20,22,25,26]. In the model for fish avoiders (model 3), approximately half of the *n*-3 LCPUFA was derived from lean red meat and half from *n*-3 LCPUFA enriched products. Interestingly, increased meat consumption has been shown to be associated with lower odds of depression [34].

Diets that exclude fish, red meat and eggs are usually lower in *n*-3 LCPUFA [35]. The plant-based *n*-3 PUFA ALA can be converted into EPA and DHA, but the conversion rate is very inefficient [36,37]. Therefore, a lacto-ovo vegetarian diet would need to include *n*-3 LCPUFA enriched products or, alternatively, take encapsulated fish oil or micro-algal oil. However, long-term consumption of encapsulated fish oil may not be feasible for many individuals due to compliance, while enriched foods have been shown to be effective in increasing LC *n*-3 intake and status [27,38]. Therefore, the food industry is encouraged to develop a wider range of enriched staple food products with higher concentrations of *n*-3 LCPUFA that include both algal and fish sources of *n*-3 LCPUFA.

It is possible to meet *n*-3 LCPUFA intake with enriched foods, but it may not be feasible or practical long-term, due to the source of *n*-3 LCPUFA and the increased cost associated with enriched foods. Some enriched foods such as the almond meal, bread and the powdered beverage drink all use tuna oil as the source of *n*-3 LCPUFA. Therefore, these products would only be suitable for vegetarians that include fish in their diets. In addition, there is the burden of increased cost associated with a diet high in *n*-3 LCPUFA enriched products, as these products are more expensive than the un-enriched varieties. Lacto-ovo vegetarians may need to take algal supplements containing *n*-3 LCPUFA in order to meet SDT due to the unrealistic goal of consuming up to 66 serves of enriched foods per week. Furthermore, a recent Australian population based study by Rahmawaty *et al.* [39] showed that replacement of actual bread, milk, egg, and yoghurt consumption with *n*-3 LCPUFA enriched varieties, doubled the *n*-3 LCPUFA median intakes in non-fish consumers, without major dietary changes [39]. This doubling of intakes in non-fish consumers, still falls far short of the SDT, and hence there is a need for a wider range of enriched foods and supplements for lacto-ovo-vegetarians that include algal sources of *n*-3 LCPUFA.

The majority of Australians with low *n*-3 LCPUFA intake are people that do not consume fish/seafood since there are more non-fish consumers than consumers who do not meet the SDT for the prevention of chronic disease [40]. Furthermore, vegans have much lower *n*-3 status compared to omnivores [35], as they do not consume preformed *n*-3 LCPUFA in their diets and solely rely on the conversion of ALA to *n*-3 LCPUFA.

In terms of cost, on average the cost of quality lean red meat is similar to that of “excellent” and “good” sources of fish (Table 3). Fish is one of the cheapest sources when expressing the cost per 100 mg *n*-3 LCPUFA. Atlantic Salmon costs approximately 29 AUD per kilogram, but only costs 16 cents per 100 mg *n*-3 LCPUFA (equivalent to 80 cents per 500 mg *n*-3 LCPUFA). Meat is on average 25 AUD per kilogram, but costs 2 AUD per 100 mg *n*-3 LCPUFA (equivalent to $10 per 500 mg *n*-3 LCPUFA), which is more than 10-fold higher than Atlantic salmon. In terms of providing 100 mg *n*-3 LCPUFA, the *n*-3 LCPUFA enriched foods are comparable to, or slightly cheaper than, lean red meat, but are more expensive when compared to fish. Australians consume at least seven times more meat than fish [18] and on average consume 160 g per day [18], which demonstrates their willingness to pay for it. Given the similarities in cost per kilogram, fish is by far the better option when assessing the amount of *n*-3 LCPUFA per cost. However Australians do not consume fish for a variety of reasons including the smell, bones, pollutants, family members not liking it, taste, the preparation and price [41]. Amongst the fish consumers, price was the main negative effect for consuming fish [40,41], however, this study clearly shows that the average cost of fish is similar to that of lean red meat, yet fish supplies 10 times more *n*-3 LCPUFA than lean red meat.

Based on Australian food culture and eating patterns, non-fish sources of *n*-3 LCPUFA are increasingly important for meeting SDT for *n*-3 LCPUFA. The modelling research highlights the difficulties in currently meeting the SDT for *n*-3 LCPUFA if you are a non-fish or low-fish consumer, as very high and regular consumption of enriched food products are required. This may not be feasible for many consumers due to cost implications, compliance issues and availability of enriched products. Therefore, there is a need for a greater variety of staple foods enriched with *n*-3 LCPUFA, such as spreads, oils, breads and cereals, to make it easier for consumers to meet the SDT.

Better use of waste in existing fisheries [42,43] will contribute to sustainable sources of *n*-3 LCPUFA and a range of new research projects are underway to provide a range of sustainable long-term dietary solutions to meet *n*-3 LCPUFA needs. This includes research on land plant sources of *n*-3 LCPUFA [44] for use as feedstock in livestock production and aquaculture and novel plant sources of *n*-3 LCPUFA [45].

The easiest way of achieving the SDT for *n*-3 LCPUFA, is the consumption of two “excellent” or “good” fish meals per week. Furthermore, in addition to *n*-3 LCPUFA, fish also contains other vital nutrients like iodine, selenium, zinc, and is a good source of protein. Hence further research is required on how to encourage more frequent consumption of fish and seafood amongst Australian consumers.

## 5. Conclusions

The SDT can be achieved for adults and children with two serves of fish containing 2000 mg/100 g *n*-3 LCPUFA per week without red meat and enriched foods. Fish avoiders who consume red meat can meet SDT recommendations via four serves of red meat/week and at least 20 serves of enriched foods per week, while lacto-ovo vegetarians need at least 37 serves of enriched foods per week. These 4 modelled diets meet the SDT for all life stages. Therefore, Australians are encouraged to meet their *n*-3 LCPUFA intake by either: an increase in fish/seafood consumption from sustainable sources, or ensure that they meet *n*-3 LCPUFA recommendations by consuming a combination of red meat, *n*-3 LCPUFA enriched products and/or fish/algal supplements. Further research is required on how to encourage more frequent consumption of fish and seafood amongst Australian consumers.

## Appendix

**Table A1:** *n*-3 LCPUFA enriched products and the non-enriched varieties.

Food Type	Full Name of Food (Including Brand Name)	Serve Size (g)	Units per Serve	Serve Unit Name	Total FAT/100 g	*n*-3 LC PUFA mg/100 g	*n*-3 Source	Pack Size	Units per Pack	Average Price $/Pack	Average Price AUD/100 g AUD/Serve
Bread	Tip Top 1 Sunblest Up Omega-3 DHA wholemeal/sandwich bread	74–78 g	2	slice	3.5	44	Tuna oil 0.3%	700 g	18	3.98	0.57/100 g
											0.45/serve
Bread	Tip Top 1 Sunblest Up Wholemeal Bread/White Sandwich	74–78 g	2	slice	3.5	N/A	N/A	650 g	18	3.99	0.57/100 g
Egg	Pace Farm Omega-3 Free Range Body Egg	102	2	egg	9.9	200	N/S	700 g	12	6.30	0.90/100 g
											0.52/egg
Egg	Pace Farm Cage Eggs	102	2	egg	9.9	N/S	N/S	700 g	12	4.62	0.66/100 g
											0.39/egg
Egg	Pace Farm Cage-Free Liberty Eggs	102	2	egg	9.9	N/S	N/S	700 g	12	5.35	0.76/100 g
											0.45/egg
Egg	Veggs for Families Organic Grain Fed Hens	104	2	egg	10.1	110	N/S	700 g	12	5.99	0.90/100 g
											0.50/egg
Egg	Farm Pride free range Omega 3 700 g	104	2	egg	9.4	110	Algal (Life’s DHA)	700 g	12	6.49	0.93/100 g
											0.54/egg
Egg	Farm Pride free range Omega 3 600 g	90	2	egg	9.15	50	Algal (Life’s DHA)	600 g	12	6.49	1.08/100 g
											0.54/egg
Eggs	Home Brand Woolworths	104	2	egg	9.9	110	N/S	700 g	12	2.69	0.38/100 g
											0.22/egg
Eggs	Coles Free Range Eggs	104	2	egg	10.0	N/S	N/S	700 g	12	5.33	0.76/100 g
											0.44/egg
Eggs	Ecoeggs	100	2	egg	10.1	230	N/S	550 g	10	6.42	0.64/egg
											1.17/100 g
Yoghurt	Vaalia “My first yoghurt” for infants >6 months—Vanilla	60	1	cup	2.7	67	DHA algal oil	60 g	6	3.79	1.05/100 g
											0.63/tub
Yoghurt	Vaalia for toddlers >12 months—Vanilla	90	1	cup	2.7	67	DHA algal oil	90 g	6	4.49	0.83/100 g
											0.75/tub
Yoghurt	Vaalia for toddlers >12 months—Peach	90	1	cup	2.8	67	DHA algal oil	90 g	6	4.49	0.83/100 g
											0.75/tub
Yoghurt	Vaalia for toddlers >12 months—Strawberry	90	1	cup	2.8	67	DHA algal oil	90 g	6	4.49	0.83/100 g
											0.75/tub
Yoghurt	Vaalia for toddlers yoghurt vanilla & peach	90	1	tub	3.1	N/A	N/A	90 g	6	4.89	0.91/100 g
											0.82/tub
Yoghurt	baby Yoplait (for ages >6 months)—Pear/Peach	100	1	tub	3.1	N/A	N/A	100 g	4	3.99	1.00/100 g
											1.00/tub
Milk	Dairy Farmers “Kids” Milk	250 mL	1	cup	3.4	120	*n*-3 DHA oil	1000 mL	4	2.69	0.27/100 mL
											0.67/cup
Milk	Pura Kids Milk	250 mL	1	cup	8.5	30	Tuna oil	2000 mL	8	4.27	0.21/100 mL
											0.53/cup
Milk	Dairy Farmers New Regular milk (2% fat)	250 mL	1	cup	2	N/A	N/A	1000 mL	4	2.24	0.56/cup
											0.22/100 mL
Milk	Farmers Best—source of Omega-3	250 mL	1	cup	1.4	13.2	N/S	1000 mL	4	2.56	0.64/cup
											0.26/100 mL
Milk	Farmers Best—Original	250 mL	1	cup	1.4	N/A	N/A	1000 mL	4	2.63	0.66/cup
											0.26/100 mL
Milk beverage powder	Boost foods Nutriboost Chocolate	27	N/A	N/A	2.1	117	refined tuna oil	430 g	16	5.80	1.35/100 g
											0.36/serve
Milk beverage powder	Boost foods Nutriboost Strawberry	43	N/A	N/A	1	117	refined tuna oil	430 g	10	5.80	1.35/100 g
											0.58/serve
Baking	Lucky Almond Meal Omega 3	14 g *	N/A	N/A	50.6	0.12	tuna oil	100 g	N/A	3.73	3.73/100 g
											0.52/serve
Baking	Lucky Almond Meal	14 g *	N/A	N/A	50.6	N/A	N/S	200 g	N/A	6.71	3.36/100 g
											0.47/serve
Juice	Berri Australian Fresh Juice Orange Extra Pulp Omega	250 mL	1	cup	<1	50	fish oil	1000 mL	4	4.63	1.16/100 mL
											1.16/serve
Juice	Berri Australian Fresh Orange Extra Pulp	250 mL	1	cup	<1	N/A	N/A	1000 mL	4	4.02	1.00/serve
											1.00/100 mL

* 14 g is the equivalent used to make one Friand based on a standard Women’s Weekly Magazine recipe. N/A—not applicable; N/S—not specified.
